# Amphitrite: A program for processing travelling wave ion mobility mass spectrometry data

**DOI:** 10.1016/j.ijms.2012.09.005

**Published:** 2013-07-01

**Authors:** Ganesh N. Sivalingam, Jun Yan, Harpal Sahota, Konstantinos Thalassinos

**Affiliations:** a Institute of Structural and Molecular Biology, Division of Biosciences, University College London, London, UK; b Institute of Structural and Molecular Biology, Crystallography, Birkbeck College, London, UK

**Keywords:** Ion mobility mass spectrometry, Travelling wave ion mobility, Software, Data processing

## Abstract

Since the introduction of travelling wave (T-Wave)-based ion mobility in 2007 a large number of research laboratories have embraced the technique, particularly those working in the field of structural biology. The development of software to process the data generated from this technique, however, has been limited. We present a novel software package that enables the processing of T-Wave ion mobility data. The program can deconvolute components in a mass spectrum and uses this information to extract corresponding arrival time distributions (ATDs) with minimal user intervention. It can also be used to automatically create a collision cross section (CCS) calibration and apply this to subsequent files of interest. A number of applications of the software, and how it enhances the information content extracted from the raw data, are illustrated using model proteins.

## Graphical abstract

**Graphical abstract:**

**Figure:**
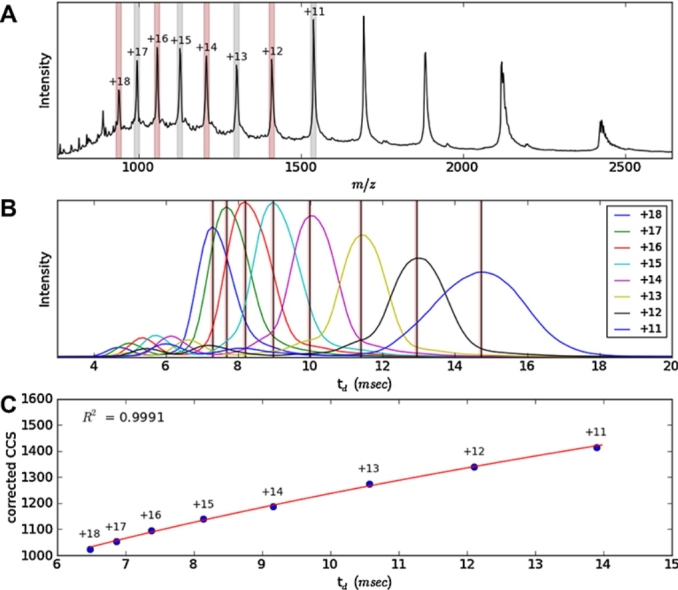

**Highlights:**

► Development of the first software to automate the processing of travelling wave ion mobility mass spectrometry (TWIM-MS) data. ► Automatic creation and application of collision cross section calibration to TWIM-MS data. ► Creation of fine-grained collision cross section vs. *m*/*z* heat maps that can be overlaid between experimental conditions.

## 1 Introduction

Ion mobility is a gas-phase technique that separates ions as they travel through a counter flowing neutral target gas under the influence of an applied electric field. The time it takes an ion to traverse the cell is related to its mass, charge, and the rotationally averaged collision cross section (CCS) of an ion [1–3]. Ion mobility coupled to mass spectrometry (IM-MS) is a powerful analytical technique that was initially only available in a few laboratories capable of building such specialised instruments. The primary means of performing IM-MS separations was based on drift cell technology [4].

Shortly after the description of a commercial instrument that was modified for IM-MS measurements [5], the introduction of travelling wave (T-Wave) ion mobility separation [6], incorporated in a commercial quadrupole time-of-flight instrument (Synapt HDMS, Waters Corp.) [7], further popularised the technique. In addition to the high mass accuracy obtainable, the Synapt can be used to carry out ion mobility-tandem mass spectrometry experiments by performing collision induced dissociation (CID) before and/or after the mobility cell. A second generation instrument, the Synapt G2, was introduced in 2011 with an up to four-fold increase in the T-Wave ion mobility resolution, as expressed in terms of Ω/ΔΩ [8], where Ω is the rotationally averaged CCS. Another attractive feature of the Synapt instruments is that they can be modified for high mass operation by the incorporation of a 32 K quadrupole, allowing the selection and transmission of high *m*/*z* species.

T-Wave ion mobility mass spectrometry (TWIM-MS) has so far been used to study a number of synthetic and biological molecules such as polymers [9–11], carbohydrates [12], peptides [13,14] and lipids [15,16]. The majority of applications, however, have been within the structural biology field as TWIMS-MS has clear advantages over other established techniques within this area. Proteins that exhibit too much conformational flexibility or that are too large to study by established techniques such as X-ray and NMR respectively, can still be amenable to analysis by means of TWIM-MS. In addition, TWIM-MS can be used to separate and study co-existing populations present in solution [17] in contrast to the majority of other biophysical techniques that can only provide information regarding the population average. TWIM-MS has been used to probe the conformation of soluble proteins and proteins bound to various ligands [18–22], protein complexes [23–26], proteins involved in misfolding and aggregation [18,27,28] intact viruses, [29,30], and membrane proteins [31,32]. In conjunction with CID, TWIM-MS has also been used to probe the structural stability of such molecules [20,33,34].

For a large number of the applications mentioned, there is no requirement to convert arrival time distributions (ATDs) to CCS in order to answer the biological question studied. Obtaining a CCS, however, is essential in cases where the CCSs are used as a way of filtering computer generated models [35–37]. Classical IM-MS instrumentation uses a drift cell mobility separation device. While the physical principles behind drift cell IM-MS are well understood and can be used to obtain a CCS for each ion studied [38], the same is not true for the T-Wave-based device. Despite initial attempts to characterise the T-Wave device, [39] the ion motion in the device is still not fully understood and cannot be used to derive CCSs directly from the arrival time (t_*d*_) data, however, a number of protocols have been developed which allow the calibration of the T-Wave against standards of known cross section [13,22,25,40]. A number of such standards are available in the form of peptides [41], proteins [41], protein complexes [42,43] and drug-like molecules [44].

Despite the advances in both TWIM-MS instrumentation development and the growing applications, advances in the software to process such data has been limited. The only software currently available is Driftscope (Waters Corp.) which involves extensive manual user interaction. A user has to identify the peaks in the mass spectrum, which can be challenging especially when dealing with spectra containing more than one components such as heterogeneous protein complexes, then use these to reconstruct the corresponding ATD. From this distribution the drift time(s) of maximum intensity are extracted for further analysis. This manual intervention can be labour intensive and can also introduce errors in the analysis. While programs to process intact protein and protein complex MS data [45–48] are appearing in the literature, there is still no program for the automatic processing of TWIM-MS data.

In this work we present a novel software for the processing of TWIMS-MS data. The software automates the deconvolution of the MS data and automatically extracts ATDs from the raw data files. It also allows for the facile creation of a calibration that can then be applied to entire data sets automatically. The software can be used to create CCS vs. *m*/*z* heat maps that can be overlaid between different experimental conditions, something that allows for a more in-depth probing of the structural changes taking place between different conditions. Having a program do these analyses allows for the standardisation of the data processing, making the entire process more objective and reproducible between different practitioners. A number of different uses of the program, with a particular focus, on commonly encountered structural biology applications are illustrated using model proteins.

## 2 Materials and methods

### 2.1 Sample sources and preparation procedures

cytochrome *c* from equine heart, myoglobin from equine heart, alcohol dehydrogenase (ADH) from *Saccharomyces cerevisiae*, bovine serum albumin (BSA), and concanavalin A from *Canavalia ensiformis* were purchased from Sigma Aldrich (St. Louis, MO). Serum amyloid P component (SAP) from human serum was purchased from CalBioChem, Merck Biosciences Ltd. (Darmstadt, Germany). For native experiments, protein samples were buffer exchanged into 250 mM ammonium acetate, and concentrated to 20 μM using Amicon Ultra 0.5 ml centrifugal filters (Millipore UK Ltd, Watford, UK). For denaturing experiments, protein samples were buffer exchanged into a 49:49:2 (v:v:v) ratio of H_2_O: methanol: acetic acid, and concentrated to 20 μM using Amicon Ultra 0.5 ml centrifugal filters.

### 2.2 TWIMS-MS

Mass spectrometry experiments were carried out on a first generation Synapt HDMS (Waters Corp., Manchester, UK) mass spectrometer [7]. The instrument was mass calibrated using a 33 μM solution of Cesium Iodide in 250 mM ammonium acetate. 2.5 μl aliquots of samples were delivered to the mass spectrometer by means of nanoESI using gold-coated capillaries, prepared in house [49]. Typical instrumental parameters were as follows unless otherwise specified: source pressure 5.5 mbar, capillary voltage 1.10 kV, cone voltage 40 V, trap energy 8 V, transfer energy 6 V, bias voltage 15 V. IMS pressure 5.18 × 10^−1^ mbar, IMS wave velocity 250 m/s, IMS wave height 6 V, and trap pressure 4.07 × 10^−2^ mbar.

### 2.3 Experimental procedures

cytochrome *c* was analysed with a bias voltage of 30 V, with denatured myoglobin being used as a calibrant for obtaining CCS. For the heating experiment ADH was incubated at 60 °C for 30 min in a heat block. The sample was removed from the heat block and immediately introduced to the mass spectrometer. Instrumental parameters were optimised as follows: source pressure 4.50 mbar, cone voltage 60 V, trap energy 15 V, transfer energy 12 V, and IMS wave height 7 V. BSA and concanavalin A were used as CCS calibrants. For the collision unfolding experiment the native fold of cytochrome *c* was disrupted by increasing the bias voltage by 10 V at a time, from 10 V, until reaching 80 V. Instrumental parameters were optimised as follows: source pressure 3.55 mbar, cone voltage 30 V, and IMS wave height 7 V. Denatured myoglobin and ADH were used as CCS calibrants. For the mixing experiment, ADH, BSA, and concanavalin A were mixed in an equimolar ratio, and the instrumental parameters were optimised as follows: trap energy 60 V, transfer energy 30 V, bias voltage 22 V. IMS wave height 7 V. SAP, BSA, and concanavalin A were used as CCS calibrants.

### 2.4 Software development

During a TWIM-MS experiment ion arrival time distributions (ATDs) are recorded by synchronizing the oa-TOF acquisition with the gated release of a packet of ions from the trap T-Wave. For each packet of ions 200 mass spectra are acquired at a rate dependent on the pusher frequency.

Amphitrite handles the data in the form of a *n* × 200 matrix (where *n* is the number of *m*/*z* bin increments), with individual vectors to describe the associated axes. This matrix can be used to generate the full mass spectrum of all arrival times by summing down to a *n*-length vector. Additional manipulations can be carried out by selecting sections of the matrix by index, for example the arrival times of a particular ion could be extracted by supplying the lower and upper *m*/*z* limits, and then summing along the *m*/*z* axis. The manipulations of this matrix forms the basis of the functionality of the program.

The software was developed using the Python programming language [50]. Several Python modules were utilised for data analysis NumPy, SciPy [51] and Matplotlib [52], and the graphical user interface was developed using wxPython [53]. The initial conversion of a raw TWIM-MS file to an Amphitrite project file can only be run under Microsoft Windows, however, all other aspects of Amphitrite are cross platform compatible and installer binaries for Linux and Mac OS X systems are available on the website http://www.homepages.ucl.ac.uk/∼ucbtkth/amphitrite.html. The software was developed on a 3.4 GHz quad-core processor machine with 16 GB memory running Ubuntu 12.04. Processing times quoted are for a 2011 MacBook Air with a 1.7 GHz dual-core processor and 4 GB memory.

## 3 Results and discussion

Until now, Driftscope (Waters Corp.) has been the sole program used to display and manipulate raw TWIM-MS data. The introduction of Amphitrite facilitates increased customisability of plots as well as the automation of previously labour-intensive, subjective and hence non-reproducible tasks. Using standard proteins we describe various examples of how the program can be used.

### 3.1 Mass spectrum simulation

Programs capable of automatic and semi-automatic analysis of mass spectrometry data of proteins and protein complexes are becoming increasingly available [45–48]. Amphitrite also includes an algorithm for the deconvolution of mass spectra, as fitting peaks to the mass spectrum is the first process in the automatic extraction of corresponding ATDs to these peaks. A Gaussian model (Eq. (1)) is used to represent the distribution of peak heights of the ion peaks within a charge state distribution of a given molecular species, and this is used as a constraint in mass spectral simulations. The individual peaks, corresponding to a specific charge state, can be modelled as Gaussian (Eq. (1)), Lorentzian (Eq. (2)) or a hybrid peak shape which consists of Gaussian and Lorentzian regions (Eq. (3)) where *A* is the amplitude, *μ* is the mean, and Γ is the full width half maximum (FWHM) of the peak.

(1) $$f(x)={\mathrm{Ae}}^{-(x-\mu {)}^{2}/2{\left(\Gamma /2\sqrt{2\ln2}\right)}^{2}}$$

(2) $$f(x)=A\frac{1}{\left[1+((\mu -x)/(\Gamma /2){)}^{2}\right]}$$

(3) $$f(x)=\left\{\begin{array}{cc} {\mathrm{Ae}}^{-(x-\mu {)}^{2}/2(\Gamma /2\sqrt{2\ln2}{)}^{2}} & :x\leq \mu \\ A\frac{1}{\left[1+((\mu -x)/(\Gamma /2){)}^{2}\right]} & :x>\mu \end{array}\right)$$

Throughout the examples presented here the hybrid peak shape was used and the model used to generate a complete charge state series is described in Eq. 4. *z*_0_ and *z*_*n*_ represent the lowest and highest charge state in the series, *A*_*z*_, *μ*_*z*_ and Γ_*z*_ represent the amplitude, mean and FWHM parameters for the charge state distribution Gaussian respectively and H^+^ is the mass of a proton. Additionally the mass has been denoted as “mass” to distinguish mass/*z*_*i*_ from mass-to-charge ratio (*m*/*z*).

(4) $$f(x)=\sum_{z_{i}=z_{0}}^{z_{n}}A_{z}e^{-\frac{{\left(\left((\mathrm{mass}/z_{i})+H^{+}\right)-\mu_{z}\right)}^{2}}{2\cdot {\left(\Gamma_{z}/2\sqrt{2\ln2}\right)}^{2}}}\cdot \left\{\begin{array}{cc} e^{-\frac{(x-\mu {)}^{2}}{2\cdot (\Gamma /2\sqrt{2\ln2}{)}^{2}}} & :x\leq \mu \\ \frac{1}{\left[1+((x-\mu )/(\Gamma /2){)}^{2}\right]} & :x>\mu \end{array}\right)$$

**Fig. 1:**
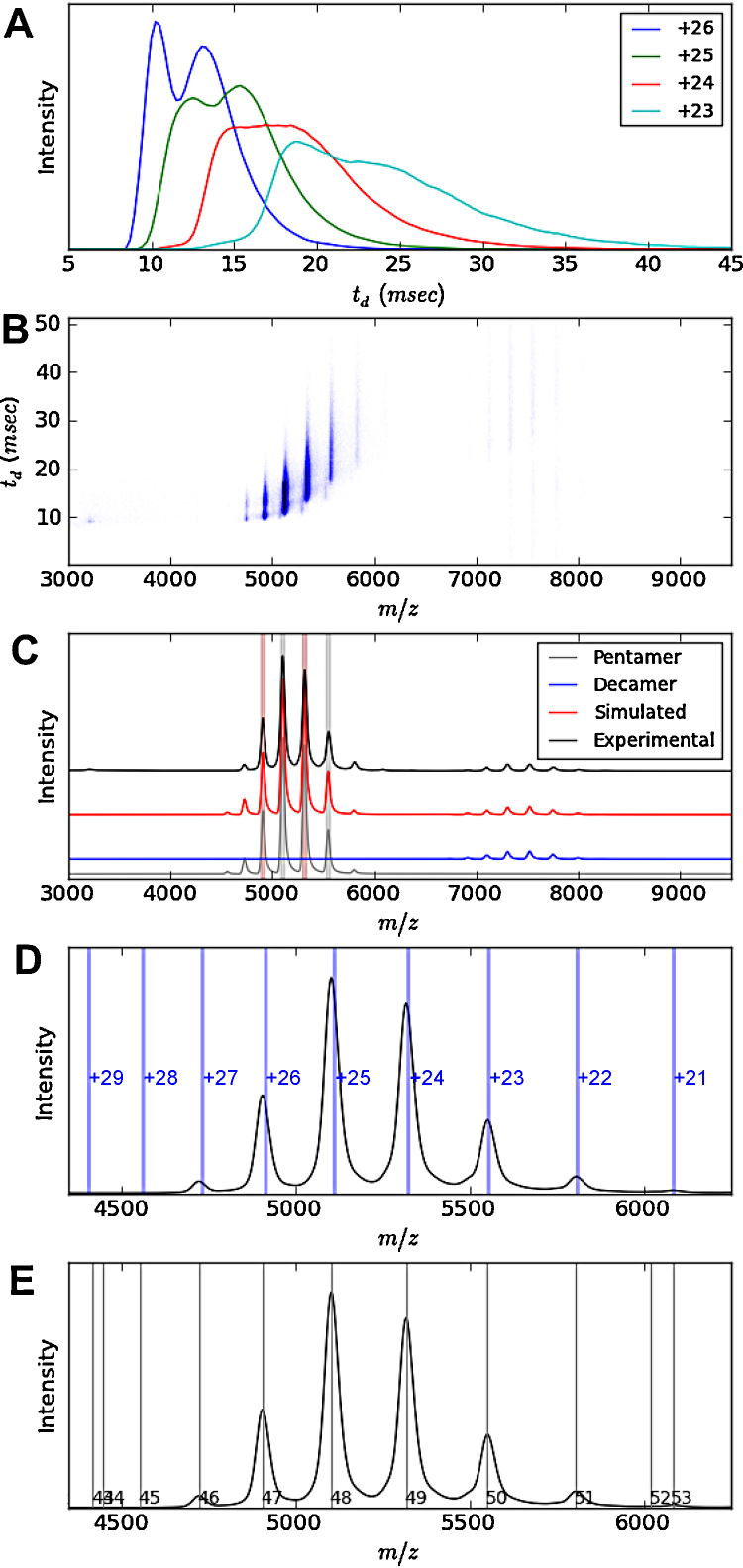
Different stages in extracting arrival time distribution plots of serum amyloid P (pentamer). The user selects peaks of the charge state series in panel E (numbers are unique peak identifiers), the mass is calculated and the theoretical charge states are then plotted over the spectrum (panel D), with the subsequent simulated spectrum plotted in panel C. Panel B shows a mass mobility plot, and using the simulated spectrum each charge state can be identified and the ATDs extracted. The ATDs have been displayed as overlaid ATD distributions for each charge state as shown in panel A.

After minimal user input the program can simulate a mass spectrum as shown in Fig. 1C with a computational processing time of under 2 s. To assess the quality of the fit an error statistic is calculated by summing the absolute error of all data points and averaging per *m*/*z* unit. In the case of Fig. 1C the error was 0.47% (of base peak intensity) per *m*/*z*. There are two ways in which one can specify the input required. If the mass of the components in the spectrum is known, it can be manually entered, along with the charge state range over which to simulate that particular mass e.g., +22 to +27 (Fig. 1D). More than one mass can be entered, and after this the program uses a least squares optimisation to minimise the difference between the simulated and experimental data. If, however, the mass of the components in the spectrum is unknown, the program aids the user in this process. The program uses a gradient method to automatically identify peaks (where *f*′(*m*/*z*) = 0 and *f*″(*m*/*z*) < 0) in the mass spectrum which are then given arbitrary unique numerical identifiers as shown in Fig. 1E.

The user then selects peaks corresponding to sequential charge state peaks of a particular species. The mass of the species is calculated using the *m*/*z* values of the peak tops. The theoretical *m*/*z* values for charge states are calculated (default 1+ to 100+) and are displayed as vertical markers along with the calculated mass and error (Fig. 1D). Both of these features help to ensure that peaks were correctly identified, as incorrect peak picking would result in misaligned theoretical charge states and large mass errors. The user then supplies the charge range to simulate, based on charge state ion peak intensities. After this process has been completed for each species, the program can fit simulated data to the supplied spectrum using least squares optimisation with the result shown in Fig. 1C. If the user then notices that a species was missed, it can be added to the simulation by following the steps described above. The data simulation algorithm can identify and deconvolve overlapping peaks and peak shoulders (see examples in Fig. 4C). An additional benefit of this feature is that it allows one to estimate the integrals of individual species with small mass differences, which can provide a more accurate measure of the peak intensity for overlapping peaks.

### 3.2 ATD extraction

Standard *m*/*z* against arrival time (t_*d*_) plots like those displayed by Drifscope can be drawn by Amphitrite (Fig. 1B) and a key improvement is the resolution of these images. In Amphitrite the user can determine the space between each data point in the *m*/*z* space i.e., how wide a particular *m*/*z* bin is. For the figures shown here a spacing of 2 *m*/*z* units was used.

Extracting ATDs across all charge states of a given spectrum has now been streamlined as the fitting procedure previously described determines the FWHM and peak centre of each of the peaks in the mass spectrum, and uses this information to automatically extract the corresponding ATD for each charge state, with the results shown in Fig. 1A.

In experiments where multiple spectra are obtained of the same protein under different conditions, the ATDs corresponding to a single charge state can be extracted for all the files in a similar manner as exemplified in Fig. 7.

### 3.3 Calibration

Protocols to convert TWIM-MS arrival times to CCS have been described previously [13,22,25,40], and Amphitrite automates this procedure, thereby reducing subjectivity that can be introduced during the ATD extraction and subsequent t_*d*_ peak selection.

**Fig. 2:**
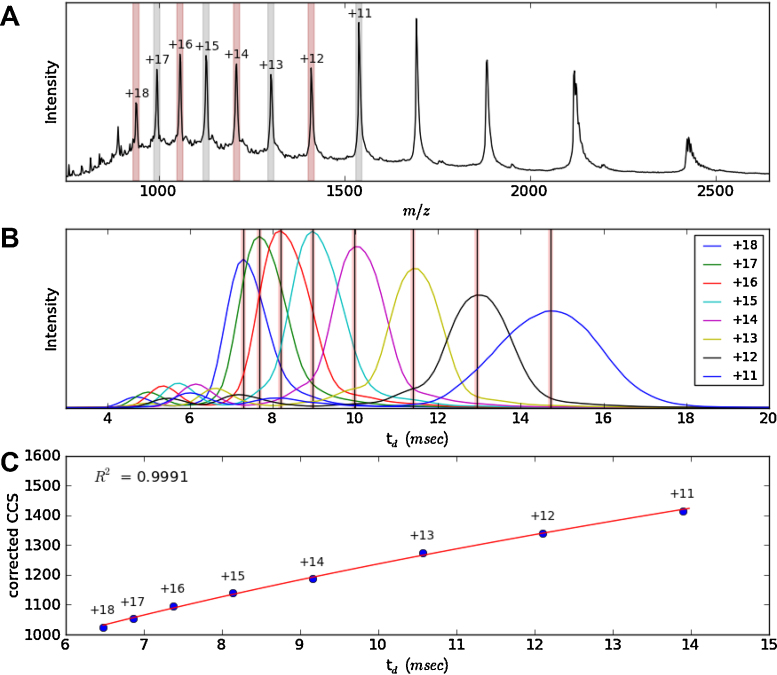
Creation of a CCS calibration using denatured myoglobin. Amphitrite automatically selects charge states (panel A), which correspond to published CCS [41]. From the selected peaks, the ATDs are extracted and plotted in panel B, and the peak tops are automatically picked and displayed. A calibration curve, using a power fit to the data, is then calculated and plotted. Poor fits can be recalculated by manually adjusting the peak tops selected in the previous stage. The calibration procedure used has been described in [13].

In Fig. 2, the process of creating a calibration is shown. From a user input perspective, the program is given the calibrant raw data file and the name of that calibrant, in this case myoglobin. Creating a calibration with more than one calibrant protein is also possible. It then automatically selects the charge states (vertical bands in Fig. 2A) that have corresponding published CCSs [41,42]. Low abundance charge state peaks can be deselected and ignored in order to improve the fit. The program automatically takes the highest intensity t_*d*_ to use in the calibration and produces the output shown in Fig. 2C. The calibration procedure used to create this figure has been published previously [13], however, alternative procedures [42] can also be calculated using Amphitrite. Outliers can be addressed by specifying alternate peak tops (which are also automatically detected), by specifically providing the t_*d*_ as input, or by removing it from the calibration.

### 3.4 Applying a calibration

The ability to read the raw data files has allowed a more fine grained approach to applying a calibration to TWIM-MS data. Since the program can identify the peaks present in the MS dimension and calculate the corresponding charge state for each peak, a calibration can be applied to each *m*/*z* “slice” rather than only to a specific t_*d*_. Here we refer to a *m*/*z* “slice” as part of the matrix that holds the extracted raw data such that *M*[*i* − *j*][1 − 200], where *i* is the lowest *m*/*z* and *j* is the highest *m*/*z* describing a peak in the MS. The calibration calculation depends on the *m*/*z*, *z* and t_*d*_ of a data point. Instead of extracting a peak and applying an overall calibration, the program recalculates the calibration for each *m*/*z* value in the dataset, which could increase the precision of the CCS values determined, as peaks with larger amounts of adduction would have the additional mass corrected for.

**Fig. 3:**
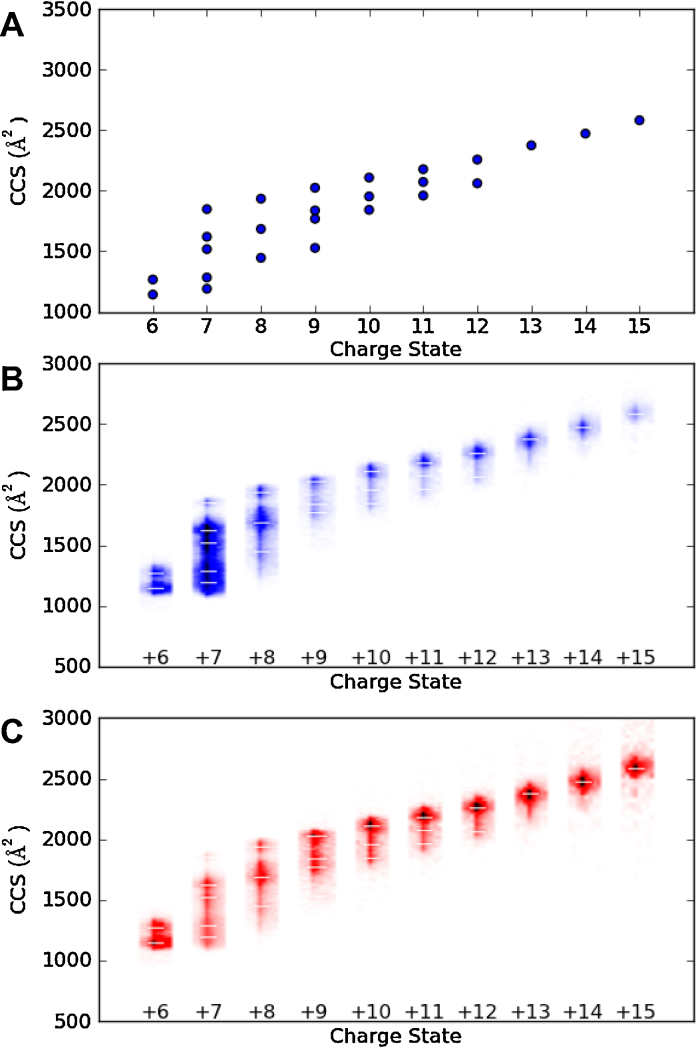
Charge state collision cross section plots of cytochrome *c*. A calibration similar to that shown in Fig. 2 was applied to the t_*d*_ data to generate the CCS data. A dot in panel A represents each peak top in the t_*d*_ for that particular charge state. The same data are shown in panel B as a heat map with peak intensity being represented by the colour intensity and the CCS of peak tops shown as a white dash. Panel C shows these data normalised by individual peak volume. (For interpretation of the references to colour in this figure legend, the reader is referred to the web version of this article.)

In Fig. 3A an archetypal IM-MS experiment result is shown, CCS vs. charge state for denatured cytochrome *c*. This is a common way found in the literature to present IM-MS data, however, a lot of the original information in the data is lost. Where more than one conformation exists for a given charge state, such as for the +8 charge state shown in Fig. 3, there is no way of deducing the relative intensities of the different conformations. It is also not possible to infer the width of each conformation in the CCS direction. Biologically, this can be very informative as an increase in the width of a CCS distribution can indicate increased conformational flexibility [27,32] and as shown recently can, in certain cases, also limit the observed ATD resolution of higher resolving instruments [37]. Comparing two proteins by overlaying figures like those shown in Fig. 3A can miss important conformational changes as the peak CCS can remain the same, while the width of each CCS can change between two conditions [27]. The figures generated by Amphitrite (Fig. 3B and C) also provide information regarding the width of the MS peak (in the *x*-axis direction) that was used to reconstruct the ATDs. The program displays the CCS dimension peak tops, as shown in Fig. 3A, and these are calculated automatically (where *f*′(Ω) = 0 and *f*″(Ω) < 0).

Features of low abundance can be visualised by using a different method of normalising the peak intensities. In Fig. 3B the colour is normalised to the intensity of the base peak in the mass spectrum, i.e., to the maximum intensity in the entire matrix holding the data, so that one can see that the +7 charge state is the most intense peak in the mass spectrum. In Fig. 3C the intensity of each charge state is normalised to the total intensity for that *m*/*z* “slice”. This allows for less abundant features to be visualised by increasing the dynamic range display for each charge state and for the conformational flexibility and adduction to be readily assessed.

### 3.5 Complex mixture analysis

**Fig. 4:**
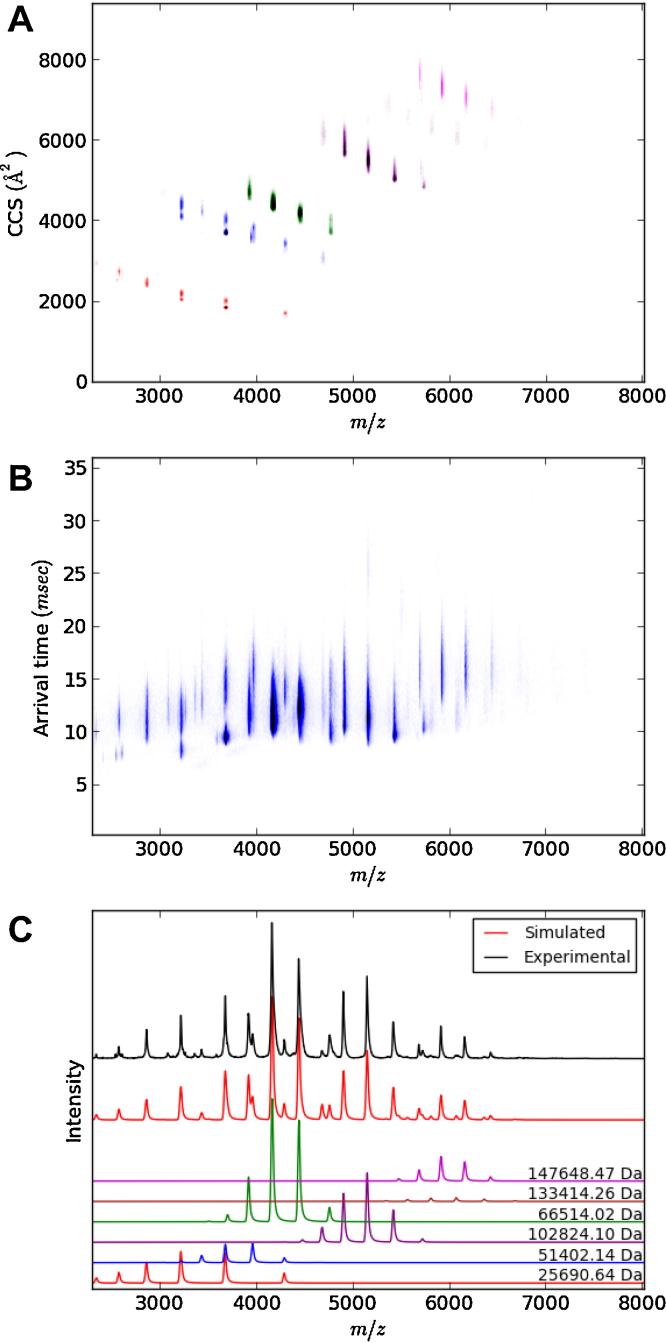
IM-MS analysis of a mixture of BSA, concanavalin A and alcohol dehydrogenase. The mass spectrum was deconvoluted into its component parts (panel C), with the raw arrival time distribution shown in panel B. Using the deconvolution data and CCS calibration (like that shown in Fig. 2), the raw arrival times can be separated and converted into CCS vs. *m*/*z* information for each molecular component (panel A). The colouring is consistent between panel A and C (concanavalin A monomer – red, dimer – blue, tetramer – purple, BSA monomer – green, dimer – brown, ADH tetramer – magenta).

By combining the deconvolution and advanced calibration features of Amphitrite, full mass spectrum CCS plots can be produced easily (see Fig. 4). To test the performance of Amphitrite when dealing with complex mixtures an equimolar mixture of bovine serum albumin, concanavalin A, and alcohol dehydrogenase was prepared.

As seen in Fig. 4 the program successfully deconvolutes the spectrum by identifying and calculating the mass and the charge state distribution parameters for all species. Additionally the individual ion peak widths are determined (Fig. 4C). A typical t_*d*_ vs. *m*/*z* plot is shown in Fig. 4B and when observed in isolation does not easily allow one to identify the number of species present. Using the parameters determined in Fig. 4C, a calibration like the one shown in Fig. 2 can be applied, transforming the t_*d*_ vs. *m*/*z* plot into a CCS vs. *m*/*z* plot (Fig. 4A). This conversion into absolute cross section separates out the individual species and results in a plot which can be more readily analysed.

### 3.6 Spectral averaging

**Fig. 5:**
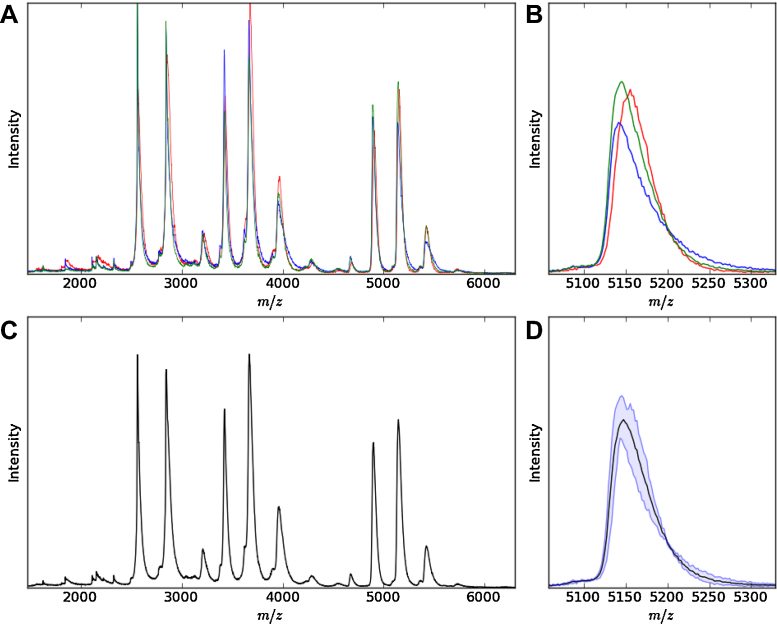
Spectral averaging. Panel A, shows the variation for the same sample measured using different capillary needles. Each individual spectrum has been overlaid and coloured differently. A peak at approximately 5150 *m*/*z* has been enlarged to portray more clearly the variation between each experiment (panel B). The average of the three spectra in panel A is plotted in panel C. The same enlarged peak in panel B is again shown in panel D, with the minimum and the maximum of the three spectra plotted as light blue lines and the mean plotted in black. (For interpretation of the references to colour in this figure legend, the reader is referred to the web version of this article.)

Collecting multiple mass spectra can help reduce the error and variation caused by certain factors such as needle-to-needle variation and needle positioning. Fig. 5A and B shows the effect of obtaining three mass spectra of the same sample using the same instrumental conditions but with three different needles. From the analysis of mass spectra of samples under different conditions (e.g., temperature), peak intensities and areas can offer information additional to the mass of the ions.

By comparing the areas of different oligomeric species under differing conditions (e.g., temperature), the formation or disappearance of oligomers in response to conditions of interest can be inferred. It is advantageous to be able to average technical replicates and compare those between conditions in order to assess whether changes are due to differing conditions rather than technical variability.

Using the program one can average spectra in the mass spectrum, t_*d*_ and CCS space. Fig. 5D shows the band between minimum and maximum intensities for a peak, with the average in the centre. The program can also be set to display the error range in terms of standard deviation, quartile ranges and percentage errors. Peak areas and heights can be automatically extracted to be used in further analyses.

### 3.7 Comparing different conditions

**Fig. 6:**
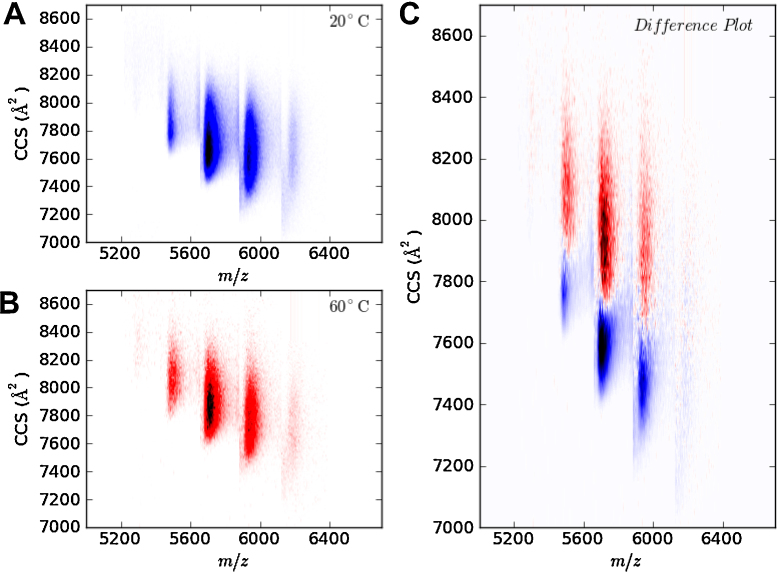
Comparison of heating experiments of alcohol dehydrogenase at 20 °C and 60 °C. Data at each temperature was replicated and averaged as shown in Fig. 5. Panel A and B show the distribution of collision cross sections for 20 °C and 60 °C respectively. A difference plot is shown in panel C, where overlapping CCS distributions in panel A and B have been subtracted from one another.

Spectral averaging was performed on the heating experiments used in Fig. 6. IM-MS can be used to monitor the effect on conformation of a discreet stressor such as heating [18]. To demonstrate this we acquired a spectrum of ADH at room temperature (20 °C), and after heating at 60 °C for 30 min. For each condition three technical replicates were acquired. In addition to the CCS vs. *m*/*z* plot of the sample at 20 °C (Fig. 6A) and 60 °C (Fig. 6B), a difference plot can also be drawn. This is shown in Fig. 6C, with the colours matching those used in Fig. 6A and B. In this figure the 20 °C and 60 °C spectra have been normalised to the volume within the plot, as this helps to make the comparison more representative. The data show that the process of heating ADH causes it to adopt a more open conformation as demonstrated by the increase in CCS.

### 3.8 Collision-induced unfolding

**Fig. 7:**
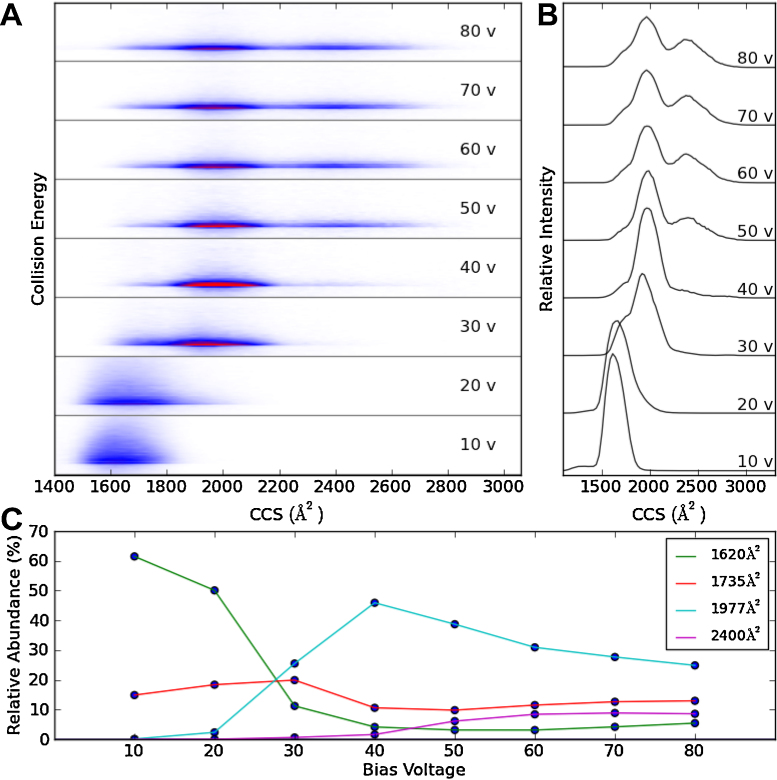
Collision induced unfolding of the 6+ charge state of cytochrome *c*. The CCS distribution of the 6+ charge state is shown in panel A as the collision energy is increased from 10 V to 80 V at 10 V increments. Intensities for panel A have been normalised to the total ion intensity for each three dimensional peak. The corresponding CCS plot for each voltage increment is shown in panel B. The relative intensity of each peak top identified from the CCS plots is also monitored as the bias voltage is increased (panel C).

A unique and very informative IM-MS experiment is gas-phase, collision-induced unfolding which can be achieved by accelerating ions into the gas filled region, prior to their entry into the T-Wave mobility cell (Fig. 7). This experiment can probe the unfolding of protein ions by monitoring changes in ATDs/CCSs upon increasing collision energies [24,33,34,54]. This experiment can be used to probe a number of experimental conditions and their effect on protein conformation/stability, however, it can generate a large number of data files as each time the collision energy is changed, a different raw data file is recorded. This is where the processing of such datasets greatly benefits from the automation offered by Amphitrite.

Fig. 7 shows the results of the collision induced unfolding for the +6 charge state of cytochrome *c*. Data were recorded at eight different collision energies. The program uses the data file which was acquired at the lowest collision energy to identify and perform a fit (in order to calculate a FWHM for that peak) to the mass spectrum as previously described in the materials and methods section. Alternatively, mass ranges can also be entered manually. The *m*/*z* range is then used to automatically extract ATDs from all files in the dataset. If a CCS calibration is provided at this stage, all ATDs are converted to CCS values. The reduced amplitude for the peaks seen for 10 and 20 V are due to the peak broadening effects of bound adducts which lessens as the collision energy is increased. This highlights the benefit of the new plot (Fig. 7A); changes in both the *m*/*z* and the ATD/CCS dimensions can be visualised simultaneously providing a means of globally monitoring ion structural changes during collision induced unfolding experiments. Finally, the program can track the peak intensities of given CCS or t_*d*_ values as shown in Fig. 7C.

## 4 Conclusions

Amphitrite substantially enhances the processing of TWIM-MS data, making the process automated and less prone to user subjectivity. It also allows for a more detailed analysis of the data acquired and the facile comparison of entire TWIM-MS datasets between different experimental conditions. A number of common applications in structural mass spectrometry were presented; we are however, planning future releases of the software which will enable application in other fields. Such an example would be to combine the functionality found in Amphitrite with available software that expedites the annotation of tandem mass spectrometry (MS/MS) data from synthetic polymers [55], to benefit researchers working in the field of polymer structure characterisation. Another area that we expect Amphitrite to have a large impact is in the processing of TWIM-MS data obtained as part of large scale proteomics experiments, as recently TWIM-MS separation has been coupled to liquid chromatography separation and a data independent mode of acquisition (MS^*E*^) [56]. It has been previously shown that in a mass-mobility plot classes of molecules such as phosphopeptides, lipids, carbohydrates and nucleotides populate different regions of such a plot [57] and Amphitrite will allow for the automatic classification of such compound classes.
